# Findings from precision oncology in the clinic: rare, novel variants are a significant contributor to scaling molecular diagnostics

**DOI:** 10.1186/s12920-022-01214-y

**Published:** 2022-03-26

**Authors:** Kenneth D. Doig, Christopher G. Love, Thomas Conway, Andrei Seleznev, David Ma, Andrew Fellowes, Piers Blombery, Stephen B. Fox

**Affiliations:** 1 Department of Pathology, Peter MacCallum Cancer Centre, 305 Grattan Street, Parkville, VIC 3000 Australia; 2 Sir Peter MacCallum Department of Oncology, University of Melbourne, Melbourne, VIC Australia

**Keywords:** Precision medicine, NGS, Clinical sequencing, Variant analysis, Precision oncology, Molecular diagnostics, Genomic database

## Abstract

**Background:**

Next generation sequencing for oncology patient management is now routine in clinical pathology laboratories. Although wet lab, sequencing and pipeline tasks are largely automated, the analysis of variants for clinical reporting remains largely a manual task. The increasing volume of sequencing data and the limited availability of genetic experts to analyse and report on variants in the data is a key scalability limit for molecular diagnostics.

**Method:**

To determine the impact and size of the issue, we examined the longitudinally compiled genetic variants from 48,036 cancer patients over a six year period in a large cancer hospital from ten targeted cancer panel tests in germline, solid tumour and haematology contexts using hybridization capture and amplicon assays. This testing generated 24,168,398 sequenced variants of which 23,255 (8214 unique) were clinically reported.

**Results:**

Of the reported variants, 17,240 (74.1%) were identified in more than one assay which allowed curated variant data to be reused in later reports. The remainder, 6015 (25.9%) were not subsequently seen in later assays and did not provide any reuse benefit. The number of new variants requiring curation has significantly increased over time from 1.72 to 3.73 variants per sample (292 curated variants per month). Analysis of the 23,255 variants reported, showed 28.6% (n = 2356) were not present in common public variant resources and therefore required de novo curation. These in-house only variants were enriched for indels, tumour suppressor genes and from solid tumour assays.

**Conclusion:**

This analysis highlights the significant percentage of variants not present within common public variant resources and the level of non-recurrent variants that consequently require greater curation effort. Many of these variants are unique to a single patient and unlikely to appear in other patients reflecting the personalised nature of cancer genomics. This study depicts the real-world situation for pathology laboratories faced with curating increasing numbers of low-recurrence variants while needing to expedite the process of manual variant curation. In the absence of suitably accurate automated methods, new approaches are needed to scale oncology diagnostics for future genetic testing volumes.

**Supplementary Information:**

The online version contains supplementary material available at 10.1186/s12920-022-01214-y.

## Introduction

Next generation sequencing (NGS) in clinical pathology laboratories for the management of patients with cancer is now routine. A number of factors have converged to allow the adoption of these technologies including the declining costs of sequencing, the replacement of narrowly focussed gene and single exon tests with assays using improved sequencing technologies that allow broader and more detailed genomic changes to be assayed. However, the use of such genomic tests has led to a significant increase in the number of variants that a laboratory must analyse to determine pathogenicity and potential diagnostic, prognostic or therapeutic use. This increasing volume of variants to be analysed has exposed a bottleneck within molecular laboratories, namely—the expert curation of variants and their integration into a clinical report. Depending on jurisdiction, curation of variants is performed by either pathologists, medical scientists or genetic counsellors following international guidelines [1, 2]. This in-house expertise represents a scarce workforce that is difficult to scale in line with variant volumes. To address this shortcoming several commercial solutions have been established that range from a complete testing service through to curation of individual variants [3–6]. Nevertheless, the variant curation bottleneck is likely to become an increasing problem and has been estimated that it will contribute to over half the cost of testing by 2026 [7].

We hypothesise that without some form of scalable artificial intelligence or other automated solution for variant analysis, the curation burden will become unsustainable. To test this hypothesis, we have examined the generation of variants over six years of genomic testing within our institution. Our aims were to (1) document the number and type of variants generated over time (2) identify which genes require the most curation effort (3) assess the benefit of commonly used publicly available variant databases and (4) compare commercial solutions to reduce the curation burden.

## Methods

All sequenced variants were uploaded to an in-house tertiary analysis decision support software system called PathOS [8] for filtering, analysis and reporting. Detailed descriptions of laboratory processes have been described previously [8]. Reported variants were manually curated using the ACMG or AMP guidelines [1, 2], to establish variant action in a patient’s clinical context. Curated variants with enriched expert annotations were deposited within a common database enabling subsequent patients presenting with the same variants to be matched to the existing variant annotations so that only novel variants need be curated. The patient’s clinical context is also stored with curated variants to inform decisions on whether the same variant appearing in a different clinical context warrants using the same stored curation or whether a new distinct, and perhaps adapted, curation of the variant and context is required. For details of the pipelines and curation workflows please refer to the Supplementary Methods section.

Patient samples were aggregated into somatic, haematology and germline sets depending on the sequencing panels used. Clinically reported variants in this study are from 453 distinct cancer associated genes (see Additional file 2: Figure S1). The genes were further broken down into overlapping categories of 63 germline genes, 401 somatic genes and 109 haematology assayed genes. These genes were categorised as either tumour suppressor or oncogene based on The Cancer Gene Census [9].

## Results

### Analysis of variants from germline, somatic and haematology assays

**Fig. 1:**
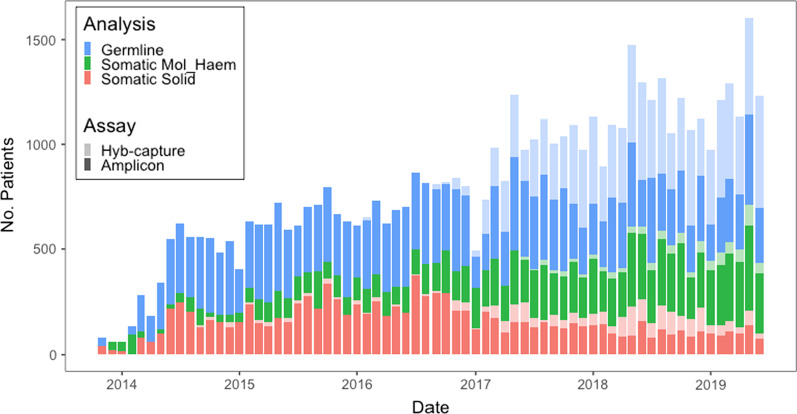
Monthly patient numbers by assay group and assay type analysed at PMCC since 2013. Amplicon assays have been used throughout while hybrid capture assays were introduced in 2017

Between the period October 2013 to May 2019, we performed next generation sequencing assays on samples from a cohort of hospital (n = 32,670) and external (n = 15,365) patients, covering a broad range of tumour streams, over a period of six years. This yielded 24,168,398 variants of which 23,255 were clinically reported from 95,954 patient samples from 48,036 patients using a heterogenous set of cancer assays (see Fig. 1). The assays were targeted cancer gene panels covering a wide range of genomic capture regions ranging from highly targeted panels of four genes through to comprehensive cancer panels of up to 701 genes. Ten different panels were employed covering varying regions of the genome using hybrid capture or amplicon technologies (see Table 1) comprising hereditary cancer germline panels, somatic panels and haematology panels for solid cancers and blood cancers respectively. A detailed breakdown by assay is provided in Additional file 1: Table S1.

**Table 1:** Breakdown of assays, panels, samples and variants contained within PMCC database (PathOS)

Analysis group	Assay	Average genes/panel (range)	Average genome coverage in Kb (range)	Patients	Samples	Average reported variants/patient (std. err.)	Average variants/patient (std. err.)
Germline	Hyb-capture	217	460.0	9283	10,728	0.3 (± 0.0)	1.4 (± 0.0)
	Amplicon	6 (4–11)	52.7 (42–81)	17,950	24,818	0.0 (± 0.0)	0.7 (± 0.0)
	Sub-total	35 (4–217)	130.3 (42–460)	27,233	35,839	0.1 (± 0.0)	1.0 (± 0.0)
Haematology	Hyb-capture	337 (312–362)	2069.6 (2052–2086)	634	1420	1.1 (± 0.1)	34.3 (± 1.2)
	Amplicon	29 (20–36)	39.2 (26–67)	9204	25,797	0.8 (± 0.0)	2.0 (± 0.0)
	Sub-total	68 (20–362)	293.0 (26–2087)	9838	27,217	0.8 (± 0.0)	4.0 (± 0.1)
Somatic	Hyb-capture	449 (90–701)	2083.7 (421–2994)	1820	3923	1.9 (± 0.1)	30.1 (± 0.5)
	Amplicon	31 (13–119)	53.0 (22–158)	9145	29,268	0.6 (± 0.0)	1.2 (± 0.0)
	Sub-total	161 (13–701)	705.3 (22–2994)	10,965	32,898	0.8 (± 0.0)	6.0 (± 0.1)
	Grand Total	96 (4–701)	404.2 (22–2994)	48,036	95,954	0.4 (± 0.0)	2.8 (± 0.0)

Of the 23,255 clinically reported variants, 17,240 (74.1%) were identified in subsequent assays and reused in reports. The remainder, 6015 (25.9%) were only observed in a single patient sample.

### Curation workload growth

**Fig. 2:**
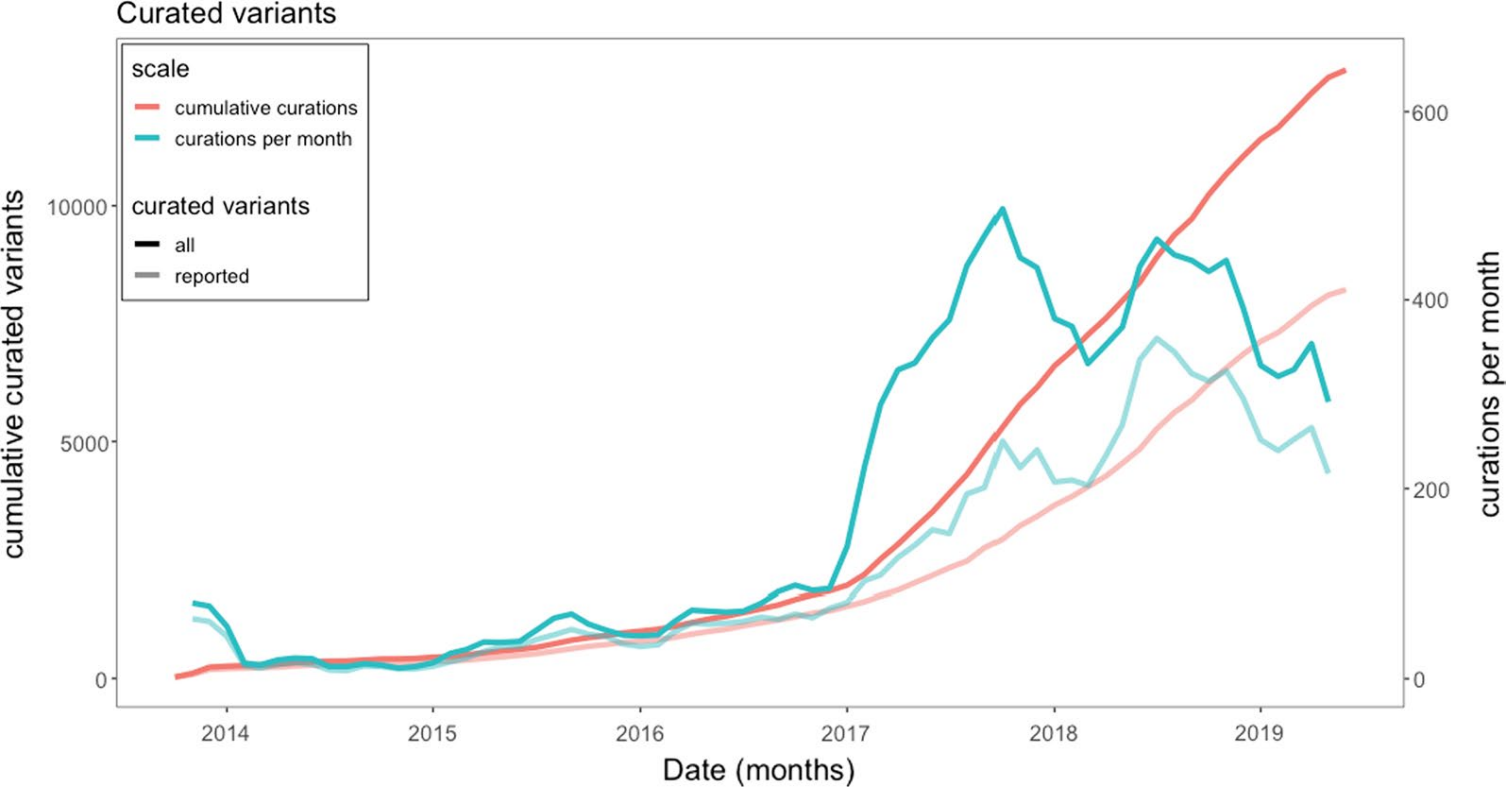
Growth of expert curated variants (3 month rolling average). The curated variants that were reported (pale lines) represent 69.1% average of all curated variants. All curated variants (solid lines) are comprised of clinically reported variants, benign, likely benign and variants of unknown significance

The total number of variants curated over the study is shown in Fig. 2 showing the significant increase with the introduction of hybrid capture assays in 2017. The solid line shows all curated variants (reported, benign and variants of unknown significance (VUS)) compared to the pale lines of reported variants (69.1% of total).

**Fig. 3:**
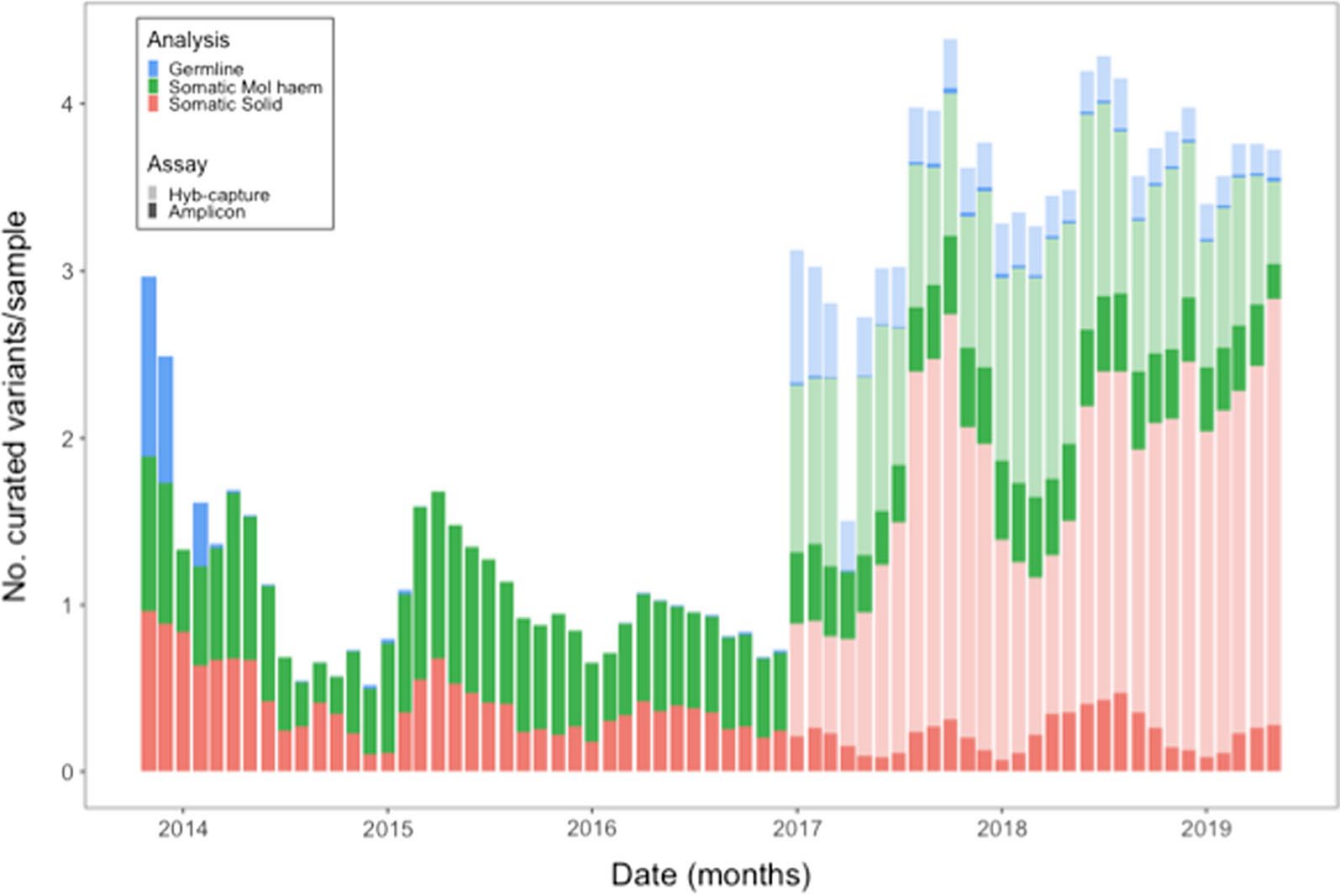
Stacked bar chart of monthly number of new variant curations per sample by analysis group and assay type (3 month rolling average). Hybrid capture technology assays were introduced at the start of 2017 (pale segments). Somatic hybrid capture variants (pink segments) dominate the reporting volume from 2017 onwards and are increasing

**Fig. 4:**
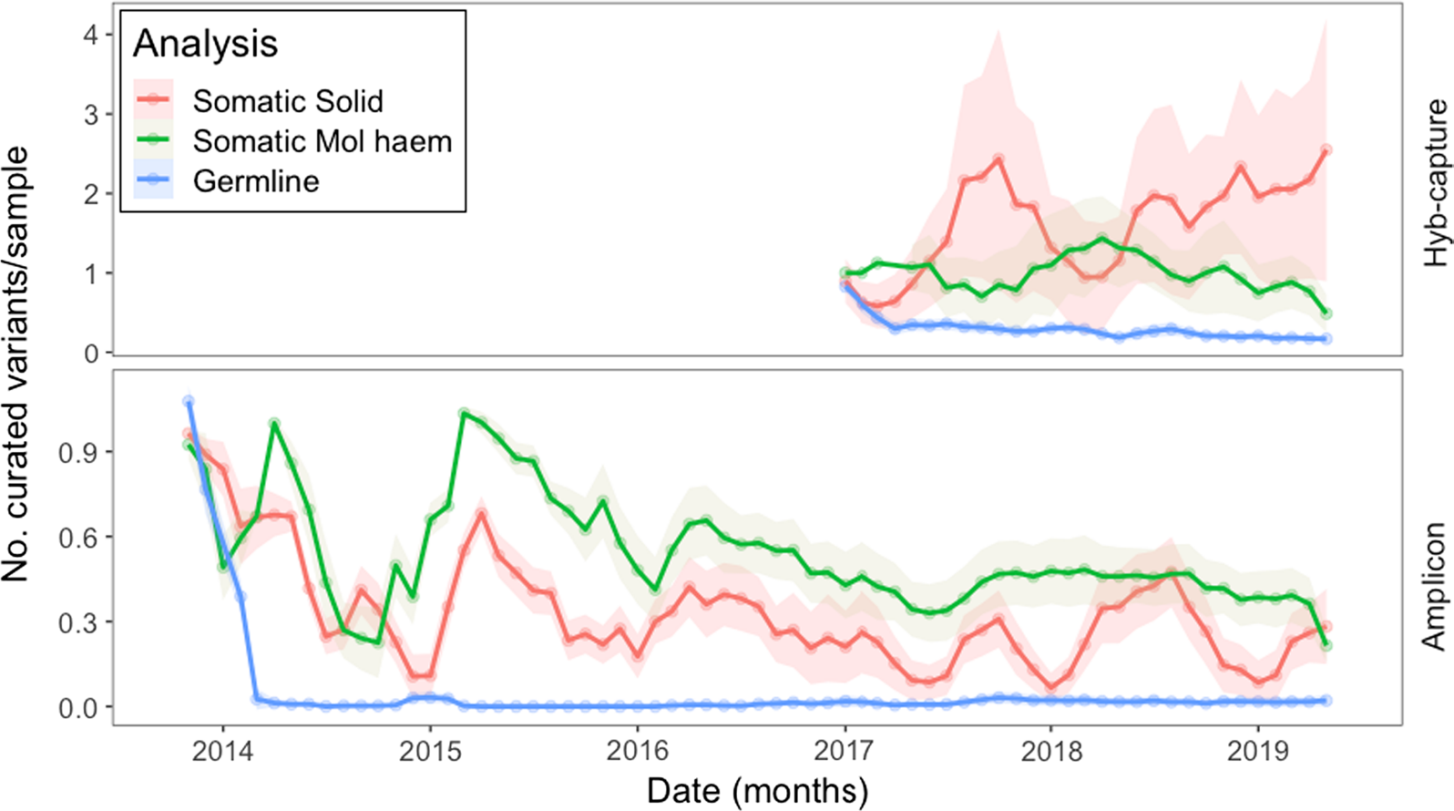
Longitudinal analysis by analysis group and assay type of mean curated variants/sample using a three-month rolling mean with 95% confidence intervals. (top chart) Hyb-capture somatic solid samples (red) are increasing in the number of new variants requiring curation. (bottom chart) Amplicon assay samples have required less than 0.5 curations each across all analysis groups over that last three years

The number of new variants requiring curation per sample per month increased from 3.38 to 3.73 from January 2017 until May 2019 (see Fig. 3). Over this period, curations of somatic hybrid capture assays rose significantly from 0.90 to 2.55 samples per month until they accounted for 68% of the curation burden per month. There was also more variability in the number of average variants per month for somatic hybrid capture assays as shown by the larger 95% confidence intervals (see Fig. 4).

### Low overlap between in-house and public databases

**Fig. 5:**
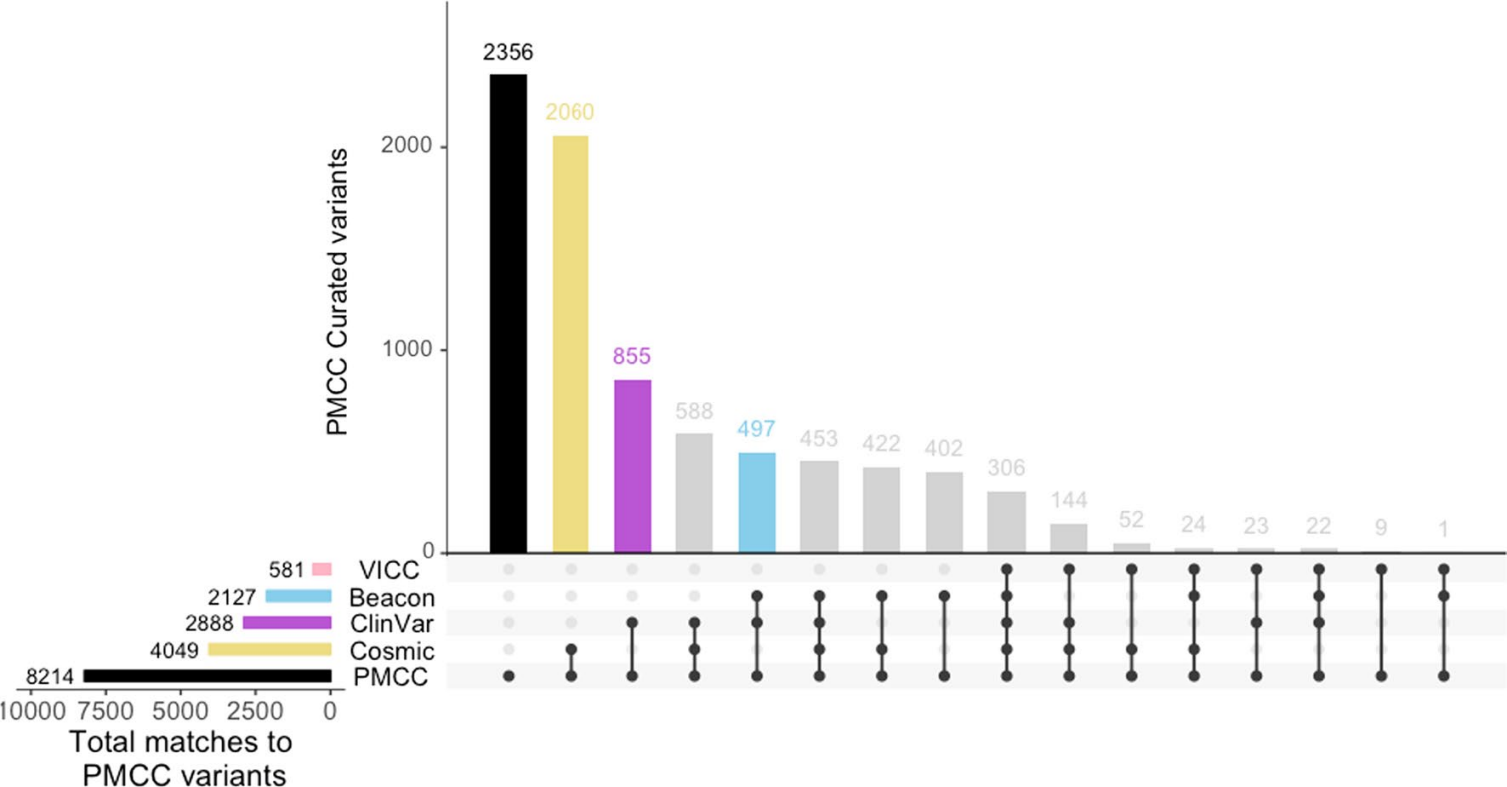
Overlap of 8214 clinically reported variants curated in PathOS with multiple public cancer variant annotation resources (COSMIC, ClinVar, VICC Meta-knowledgebase, The Beacon network). 2356 variants did not match any resources and appear novel

We compared the presence of reported variants with a number of common public genomic knowledgebases. Of the 8214 unique clinically reported variants within our in-house database, 28.6% (n = 2356) were not present within key public cancer variant resources; COSMIC [10] (size = 11,453,569 coding mutations), ClinVar [11] (size = 789,593 variants), VICC [12] (incorporating CiVIC [13], size = 2528 variants) and GA4GH Beacon network [14] (see Fig. 5). The highest number of in-house (PathOS) variant matches was to COSMIC, 4049 (49.2%), followed by ClinVar matches with 2888 (35.1%), but only 581 (7.1%) matched VICC variants. Variant matches to resources on the Beacon Network were 2127 (25.9%). Our clinically reported variants include prognostic and diagnostic variants in addition variants with a clear therapeutic option which is a focus of VICC. Further, the variants within PathOS but not present in VICC are enriched for TSGs as these variants are often loss of function variants (see Additional file 2: Figure S2 and Figure S3).

**Fig. 6:**
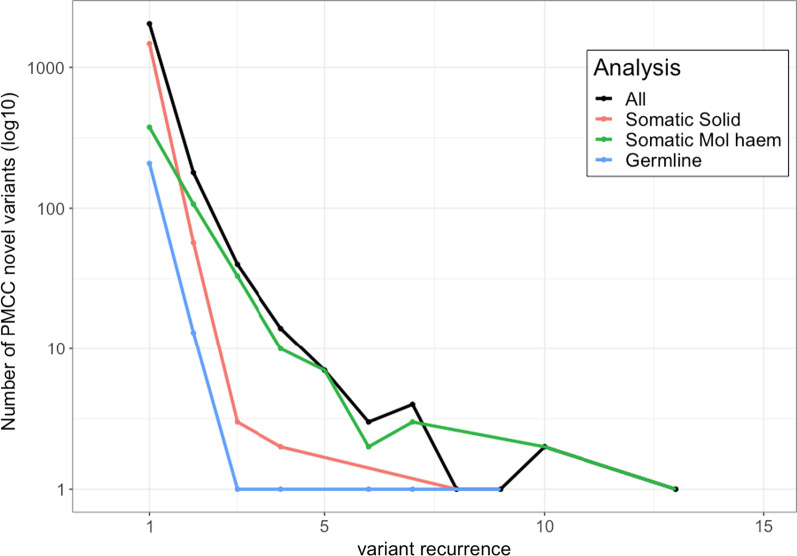
Recurrence of novel variants (n = 2356) within Peter MacCallum Cancer Centre patient samples. All variants (black) are further broken down into germline (blue), haematological (green), somatic (red) analyses. The plot highlights the majority of variants are not recurrent (n = 2041) and mostly from somatic analysis

We then examined the variants (n = 2356) not found in external knowledgebases to more closely identify their characteristics. The majority of variants (87.6%: n = 2041) were non-recurrent, that is, only reported in a single patient (see Fig. 6). Somatic assays contributed 65.5% (n = 1543), 24.8% (n = 585) from haematology assays, and 9.7% (n = 228) from germline assays. The category of variants without external knowledgebase data were curated de novo and stored in our internal database, where they provided little benefit for future patients due to the large proportion that did not reoccur within other cancer patients over the study period.

Of the in-house only variants, 43.2% (n = 1017) were from somatic assays, of missense consequence and classified as VUS (see Additional file 2: Figure S4). Analysis of gene type shows a large number of the variants were missense VUS from oncogenes (n = 239), tumour suppressor genes (n = 290), or within genes not listed in the Cancer Gene Census (n = 381) (see Additional file 2: Figure S5).

**Fig. 7:**
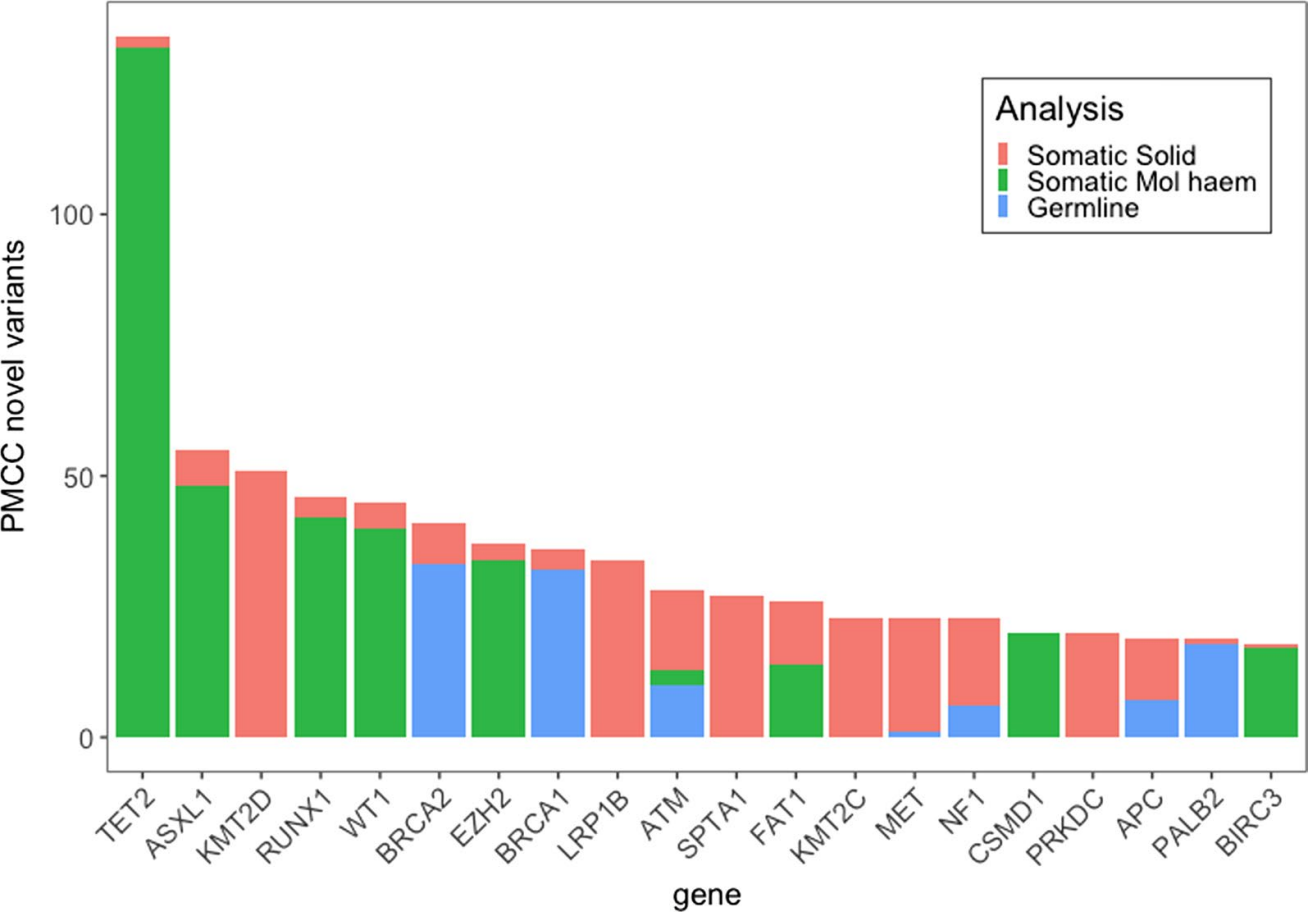
PathOS only variants by gene and analysis type (top 20 shown)

A gene level analysis of the in-house only curated variants reflects the mix of genes in our custom targeted gene panels (see Fig. 7). Key genes associated with haematological cancers contribute significant numbers of in-house only variants. In particular, the tumour suppressor TET2 is implicated in haematological malignancies [15] and 134 TET2 unique variants were reported, none of which were seen in external databases. Other genes frequently mutated in haematological malignancy included ASXL1 [16], RUNX1 [17] and WT1 [18]. This may be attributed to the large number of haematology assays within PathOS and the underrepresentation of haematological genes within the compared public resources.

### Commercial systems may increase misclassification risk

A subset of novel in-house only curated somatic and germline variants (n = 307) were submitted to a commercial tertiary analysis platform (CTAP) for annotation and pathogenicity assessment. The CTAP only used ACMG classifications for both germline and somatic variants. Although this framework is not a relevant categorisation for somatic variants, these were compared to our in-house classifications that were mapped to ACMG categories.

The subset comprised four pathogenicity classes using the ACMG classifications (‘benign’ n = 2, ‘VUS’ n = 249, ‘likely pathogenic’ n = 18 and ‘pathogenic’ n = 38). Although 81.1% (n = 249) variants were concordant for pathogenicity, 18.9% (n = 58) were discordant (see Table 2). Discordant classifications included 29 classified as ‘VUS’ by CTAP but ‘pathogenic’ by PathOS and 17 variants classified as ‘VUS’ by CTAP but ‘likely pathogenic’ by PathOS (see Additional file 2: Table S2). Of these 29 discordant classifications, 17 were non-synonymous, 11 nonsense non-synonymous and one within a splice site; 15 were substitution variants and 14 were insertions.

**Table 2:** Comparison of variant classifications between a subset of novel PathOS variants submitted to a commercial tertiary analysis platform showing concordance

CTAP	PathOS				
	Benign	Likely benign	VUS	Likely pathogenic	Pathogenic
Benign	0	0	0	0	0
Likely benign	1	0	0	0	0
VUS	1	0	248	17	29
Likely pathogenic	0	0	1	1	9
Pathogenic	0	0	0	0	0

A particular example is chr1:g.45799193dup (HGVSc:NM_001128425.1:c.240dup, HGVSp:NP_001121897.1:p.(V81Cfs*12)) classified as pathogenic due to a frameshift resulting in a stop codon leading to loss of function in the tumour suppressor MUTYH [19] but CTAP has this annotated as VUS. Another example is chr16:g.23641608T > A (hgvsc: NM_024675.3:c.1867A > T, hgvsp: NP_078951.2:p.(Lys623*) which we predicted as a truncated PALB2 protein by approximately 46%, resulting in loss of significant functional domains. Literature suggests ovarian, breast and other malignancies with a loss of HR proteins, including PALB2, have been shown to confer clinical sensitivity to PARP inhibitors and platinum agents [20–22]. CTAP had this variant classified as VUS which may lead to potential therapeutic approaches for the patient being missed.

### Comparison of gene distributions by tumour stream

From the 10,965 somatic assay patients, 3939 variants were curated according to the clinical context reported with the patient sample. The top ten clinical contexts with the most variants show that these variants are dominated by VUS classifications (see Additional file 2: Figure S6).

To examine the concordance at the gene level between databases in specific clinical contexts, we compared the top 20 genes across melanoma, colorectal and hematological malignancies in our in-house knowledgebase (PathOS) to COSMIC and ICGC by matching the primary tumour site (see Additional file 2: Figure S7). The patient gene counts were positively correlated for the melanoma (ICGC: Pearson’s r = 0.80, *p* < 0.01; COSMIC: r = 0.81, *p* < 0.01) and also for colorectal (ICGC: r = 0.74, *p* < 0.01; COSMIC: r = 0.81, *p* < 0.01) cohorts (see Additional file 2: Table S3). In contrast, the haematology stream shows marked difference in gene distributions and did not show a significant association with ICGC but did show a weak correlation with COSMIC (r = 0.63, *p* < 0.01). This may be attributed to the custom gene panels of the PMCC haematology assays and differing ranges of blood cancers incorporated into ICGC and COSMIC analysis.

## Discussion

This study conducted a longitudinal examination of clinically reported variants to assess the current and future curation workload and burden. The curation burden has become a key limitation to the scalability of genomic testing as current practices rely on the time and expertise of skilled genomic scientists to manually process the variants observed through NGS. The scalability covers the dimensions of numbers of patients assayed and the size of the genomic regions observed per assay or both.

This analysis has shown a long-term upward trend in patient numbers as well as the size of the genomic regions assayed. Both factors have resulted in an increasing number of curated variants over the study period. The in-house caching of expert curated variants should ideally have the effect of needing to curate less variants over time as fewer and fewer novel variants are seen for each assay type. This is indeed the case for all the assay groups except for somatic assays in which we show novel variants are growing over time. The germline assay group is primarily used for screening a limited number of hereditary cancer genes. This together with multiple rich publically available databases built over many years of testing yield fewer reportable variants per patient. In contrast, the somatic and haematology assays are primarily clinician requested assays for patients presenting with cancer. The rapid adoption of clinical testing of somatic cancer implicated genes has contributed significantly to the curation effort required for these assays.

The grouping of assays into germline, somatic and haematology reflects the differing curation requirements between the groups. Both somatic and haem. groups must also allow for tumour purity and clonality and so analyse a greater number of variants at much lower allele frequencies and also distinguish between germline and somatic variants. Each group has distinct but overlapping gene sets with their own pathways and mechanisms (see Additional file 2: Figure S1). The specialisation of genetic scientists into these groups adds further pressure on the availability of trained curators.

Ideally, a set of global genomic variant knowledgebases would reduce the duplication of curation effort across laboratories (whose data is frequently unshared) while also harmonising classifications across knowledgebases [23]. Although this goal has not yet been realised [24], there are active efforts by the Global Alliance for Genomic Health (GA4GH) to create such resources [25]. A meta-knowledgebase has been developed by the Variants In Cancer Consortium (VICC) that has aggregated and harmonised six different cancer variant interpretation knowledgebases, including CIViC, to collect actionable clinical interpretations for cancer associated variants [12]. An alternate model is the web-accessible Beacon Project [14], which allows aggregation of evidence for a given variant from over 100 variant resources [26, 27]. From a clinical utility perspective, different annotation resources can be ranked according to curation value offered (see Additional file 2: Figure S8). Manually curated resources such as CIViC [13] often provide the most reliable annotations and the highest clinical value, if from a trusted curator, however due to the effort required to accurately curate knowledge about a variant, these resources are limited in size. Observational resources e.g. ClinVar and COSMIC provide greater variant numbers but provide significantly less detail and less clinical benefit [10, 11]. There are also increasing numbers of national level curation databases which aggregate variants from multiple laboratories under a common framework [28, 29] as well as gene and disease specific databases such as ENIGMA [30] and IARC TP53 [31]. These inititives often provide a staging database which feeds into the larger consortium databases such as ClinVar and COSMIC.

We examined the extent to which public knowledgebases (COSMIC, ClinVar, VICC or GA4GH Beacon) and a commercial package could assist with expert curation by matching in-house clinically reported variants with external resources. We showed that at best, 71.4% of our variants were also catalogued externally. The overlap between our in-house variants and the external knowledgebases varied widely from COSMIC (49.2%), followed by ClinVar (35.1%), while only (7.1%) matched VICC variants. The low number of variants matching in VICC is likely due the therapeutic focus of the VICC knowledgebase in contrast to the other data sources. As a molecular diagnostic lab, prognostic and diagnostic variants need to be reported in addition to the therapeutically actionable variants.

These external data sources provide some assistance to our internal curation effort but by no means replace the work needed to create a complete and trusted in-house curation entry that complies with laboratory SOPs and accreditation standards. Consistancy of external knowledgebases is also a problem when incorporating external variants into in-house reports. A recent study has highlighted the difficulties in achieving consistent classifications of variants across commercial knowledgebases and also reflected the variability in ascribing clinical actionability to variants [32]. Similar variability was also found between N-of-One, IBM Watson for Genomics and OncoKB in a a study by Katsoulakis, et al. [33]. These issues will mitigate some of the benefits of public knowledge bases until there is a shared trust of the data and a common framework for variant sharing [12].

Analysis of the 28.6% of in-house only variants shows them to be mostly seen in a single patient and are enriched, relative to the set of reported variants, for indels and tumour suppressor genes. This characterisation is not unexpected as they often represent loss of function (LOF) variants in tumour suppressor genes [34] that can be commonly disrupted by indel and splice junction variants but are non-recurrent in other patients. In contrast, gain of function (GOF) variants are typically focussed at a hotspot locus [34] and well documented in therapeutically focussed public knowledgebases if actionable.

This study has shown the widespread use of variant knowledgebases by laboratories has limitations for the scalability of clinical diagnostic sequencing. This is the case even with a trusted in-house variant database which has been built up over many years or public genomic resources which are not yet comprehensive enough or sufficiently standardised to augment or replace in-house curated resources. Even when observed variants are matched with public resources, effort is needed to take external variants and apply laboratory SOPs and accreditation standards prior to reporting and storing them as a trusted in-house entry. Further, there will always be classes of variants, such as loss of function variants, that are not commonly recurring and often won’t find their way into public resources. These variants still require expert analysis of their consequences within a patient’s clinical context although the clinical information about them may be scarce.

Sophisticated computational algorithms arguably have the greatest potential to relieve the variant curation bottleneck. There are currently a large number of pathogenicity prediction algorithms available but these software need to be applied with caution due to their high false positive rate and confounding data used to train some of the algorithms [35, 36]. This is recognised by the ACMG guidelines for germline variants and AMP guidelines for somatic variant curation by specifying pathogenicity predictors must only be applied as *supporting evidence* in variant classification [1]. A detailed comparison of pathogenicity prediction tools may be found in Suybeng et al. [37]. Machine-learning approaches such as natural language processing to train curation models from medical literature, and deep-learning methods for variants may provide greater value in increasing the throughput of clinical variant interpretation, and perhaps provide the greatest hope in relieving the curation bottleneck [38, 39].

## Conclusion

This study demonstrates the challenges faced by clinical cancer genomics laboratories to efficiently deliver clinical genomic reports in the face of an increasing variant curation workload. Our work highlights that, particularly for somatic analysis, increasing the genomic coverage for clinical reporting can increase the curation workload and a large percentage of the newly identified variants will be absent from variant resources and require greater curation effort. Further, particular classes of variants, such as loss of function variants in tumour suppressor genes and private patient mutations do not appear recurrently in patients and their curation has little chance of reuse for subsequent patients. Although this study is from a large public cancer hospital, it is anticipated that genetic analysis in complex diseases other than cancer will involve many of the same issues and limitations described here. As personalised medicine is more widely adopted with greater sample numbers and larger genomic regions interrogated, we will have to be more reliant on developments in computational methods facilitating more automated approaches.

## Supplementary methods

This section covers details of additional methods used in the study.

PathOS annotates variants from sequencing pipelines and presents them within a web application for pathogenic classification prior to its generation of a clinical report. For this study we only consider SNVs and short indels. Although PathOS contains copy number variant data derived from off target alignments [40], this has only been captured across a subset of the cohort so not included for analysis. To identify relevant variants from the patient samples, sequenced samples are aligned to the GRCh37 reference genome and variants called using GATK best practice pipelines combined with in-house variant calling software [41, 42]. The called variants are normalised and 3’ shifted using custom software [8] and Mutalyzer [43] and annotated using Variant Effect Predictor [44] and other sources to enrich annotation for variant curation and pathogenicity classification. A single curated Refseq transcript is selected as representative of the variant locus and used as a basis for consistent calling of variants within gene coding regions. All sequenced samples are quality assessed using FastQC [45] and variants are filtered for common sequencing artefacts. Curated variants are classified according to ACMG or AMP guidelines for pathogenicity by experienced molecular post docs with specialisation in cancer biology using laboratory SOPs adhering to accredited standards including ISO15189.

Of the variants analysed, curated and stored in PathOS, 69.1% (n = 23,255) were clinically reported (see Fig. 4). The burden of curation remains as high for the non-reported variants (typically VUS, likely benign and benign) to ensure that they are not false negatives for diagnostic reporting.

In this analysis, the PathOS variant data were compared against four publicly available variant databases COSMIC, ClinVar, GA4GH beacons and VICC. Variants in normalised HGVSg nomenclature were compared to those present within VICC knowledgebase, queried on 1st September 2019 [12]. ClinVar variants were downloaded on 6th September 2019 and matched on HGVSg. COSMIC [10] variants were downloaded on 11th July 2019 and matched based on HGVSp position and reference allele, not the alternate allele to maximise matching. The Beacon network [14] was also queried for presence of PathOS reported variants via the web service on 1st October 2019 using HGVSg position. If a variant was identified from ClinVar/COSMIC/VICC/Beacons, but not identified through matching the data downloads from the individual resources, the matching variants were consolidated. Beacons serving computationally derived datasets such as CADD [46], or aggregators of computationally derived datasets (i.e. dbNSFP [47]) were filtered out as they over estimate presence of the variant without the ability to assess its validity. A subset from PathOS variants with no matches in the previously described public resources were submitted to a well-known commercial tertiary analysis platform to assess the value such resources can provide in variant annotation and pathogenic assessment.

Amplicon based assays (n = 36) were used throughout the study period and genomic coverage for these assays ranged from 21.9 kilobases (Kb) through to 158.2 Kb. Hybrid capture assays (n = 8) with larger genomic coverage (421.8–2994 Kb) from 90 to 701 genes (see Table 1, Additional file 2: Figure S9) are replacing the amplicon assays over time. When creating Table 1, duplicate patient samples (n = 80) occurring in multiple analysis groups and assays were excluded from the patient and average counts, to prevent biasing the analysis.

Data analysis and linear modelling were conducted using R 3.5.1. MannKendall tests were conducted using the R package Kendall. Beta coefficients between models were compared by computing a Z-score to test for equality and rejecting at 5% level of significance if there was a difference in the coefficients. To forecast the number of curations per sample per month the Holt-Winters exponential smoothing with trend and without seasonal component was applied [48] using the HoltWinters function in R stats package [48]. A two-sample test of equality of proportions with continuity correction was applied to compare percentages using the R function prop.test [49]. Analysis of variance between groups was conducted using R stats package. Comparisons between mean values were performed with a two-tailed Student’s *t*-test. A *P* value of less than 0.05 was considered statistically significant.

## Supplementary Information

**Additional file 1. Table S1**: Excel worksheet of panel attributes.

**Additional file 2. Table S2**: 24 variants with discordant pathogenicity classifications between CTAP and PathOS. **Table S3**: Correlation analysis of variant recurrence at the gene level between PathOS and publicly available datasets from ICGC and COSMIC, using Pearson’s Correlation Coefficient and log2 scale. **Figure S1**: Breakdown of numbers of genes in common across each analysis group and assay. (a) all genes surveyed in amplicon assays, (b) all genes surveyed in hyb-capture assays, (c) genes containing clinically reported variants only across all assays. (d-f) Displays the genes in common between assays (amplicon and hyb-capture) by analysis type. (g-i) Displays the genes containing reported variants in common between assays by analysis types. **Figure S2**: A gene level comparison of consequence between PathOS and VICC. The first column shows the top 20 genes in PathOS. The top row shows the genes coloured by ONC/TSG classification and the black diamond shows the number of distinct variants seen for each gene. The oncogenes have few distinct variants while TSGs and ONC/TSGs have many variants occurring in the gene. This highlights the focal nature of oncogene mutations. The third column shows varinats seen in PathOS after removing corresponding VICC variants showing that common oncogenes appear in VICC but far fewer TSGs and ONC/TSGs. **Figure S3**: These graphs show compare the top 20 variant loci between PathOS and VICC. The third graph shows the top 20 variant loci in PathOS after removing matching VICC variants. The predominance of TSG genes becomes apparent. **Figure S4**: Breakdown of novel variants not matching public cancer variant annotation resources by analysis type (n = 2,356). Each variant is classified by functional consequence and coloured by pathogenicity level. Note the high number of somatic, missense, VUS variants. **Figure S5**: Breakdown of PathOS only variants not matching public cancer variant annotation resources by analysis type (somatic, haematological and germline) (n = 2,356). Each variant is classified by functional consequence and coloured by pathogenicity level and separated based on classification of oncogene or TSG. **Figure S6**: Barplot of somatic solid variants curated by clinical contexts with > 100 variants. CUP=cancer of unknown primary, NSCLC=non-small cell lung cancer, MEL=melanoma, OVCA=ovarian cancer, PCA=prostate cancer, CRC=colorectal cancer, TCC= urothelial carcinoma, SARC=sarcoma. **Figure S7**: Comparison of patient counts by gene of reported variants between in-house database (PathOS), COSMIC and ICGC for the patient clinical contexts of melanoma, colorectal and haematological malignancies. **Figure S8**: Variant interpretation resources are not all considered equal from a somatic variant curation perspective. Resources with higher curation offer more value than observational or computationally derived resources. Figure S9: Patient samples analysed per month. A large increase in germline analysis can be observed when hyb-capture assays were implemented in 2017. There is also a steady increase over time in somatic molecular haematology (Mol_haem) samples across both assays. The number of somatic solid samples has remained relatively consistent since 2014 but an increasing number of samples were analysed with hyb-capture assays since 2017. Plotted values are calculated using a three-month rolling average.

## Data Availability

Anonymised curation data for germline assays is available through Australian Genomics (https://www.australiangenomics.org.au/tools-and-resources/shariant, https://shariant.org.au, accession: Filter: Molecular Diagnostic Path. PeterMac). The following data sources were accessed between July 2019 and June 2020 for analysis: COSMIC (https://cancer.sanger.ac.uk/cosmic/download, accession: CosmicCompleteTargetedScreensMutantExport.tsv.gz), ClinVar (https://ftp.ncbi.nlm.nih.gov/pub/clinvar/vcf_GRCh37/archive_2.0/2019/, accession: clinvar_20190902.vcf.gz), Beacon (https://beacon-project.io/, accession: Beacon of beacons was queried via the API on 1st October 2019 (https://beacon-network.org/#/developers/api/beacon-network)), VICC (https://cancervariants.org/, accession: see section ‘Data availability’ in Ref [12]) and ICGC (https://dcc.icgc.org/, accession: ICGC Data Portal Release 25).

## Abbreviations

ACMG: American College of Medical Genetics and Genomics
AMP: Association for Molecular Pathology
CADD: Combined annotation dependent depletion
CIViC: Clinical interpretation of variants in cancer
COSMIC: Catalogue of somatic mutations in cancer
CTAP: Commercial tertiary analysis platform
GA4GH: Global alliance for genomic health
GOF: Gain of function
HGVS: Human Genome Variation Society
ICGC: International cancer genome consortium
LOF: Loss of function
NGS: Next generation sequencing
SNV: Single nucleotide variant
SOP: Standard operating procedure
TSG: Tumour supressor gene
VICC: Variant interpretation in cancer consortium
VUS: Variant of unknown significance
