# Supplementary material for: Findings from precision oncology in the clinic: rare, novel variants are a significant contributor to scaling molecular diagnostics
Source: BMC Med Genomics. 2022 Mar 26;15:70. doi: 10.1186/s12920-022-01214-y (PMC8962530; doi:10.1186/s12920-022-01214-y)
Supplement: Supplementary file 2 — Additional file 2. Table S2: 24 variants with discordant pathogenicity classifications between CTAP and PathOS. Table S3: Correlation analysis of variant recurrence at the gene level between PathOS and publicly available datasets from ICGC and COSMIC, using Pearson’s Correlation Coefficient and log2 scale. Figure S1: Breakdown of numbers of genes in common across each analysis group and assay. (a) all genes surveyed in amplicon assays, (b) all genes surveyed in hyb-capture assays, (c) genes containing clinically reported variants only across all assays. (d-f) Displays the genes in common between assays (amplicon and hyb-capture) by analysis type. (g-i) Displays the genes containing reported variants in common between assays by analysis types. Figure S2: A gene level comparison of consequence between PathOS and VICC. The first column shows the top 20 genes in PathOS. The top row shows the genes coloured by ONC/TSG classification and the black diamond shows the number of distinct variants seen for each gene. The oncogenes have few distinct variants while TSGs and ONC/TSGs have many variants occurring in the gene. This highlights the focal nature of oncogene mutations. The third column shows varinats seen in PathOS after removing corresponding VICC variants showing that common oncogenes appear in VICC but far fewer TSGs and ONC/TSGs. Figure S3: These graphs show compare the top 20 variant loci between PathOS and VICC. The third graph shows the top 20 variant loci in PathOS after removing matching VICC variants. The predominance of TSG genes becomes apparent. Figure S4: Breakdown of novel variants not matching public cancer variant annotation resources by analysis type (n = 2,356). Each variant is classified by functional consequence and coloured by pathogenicity level. Note the high number of somatic, missense, VUS variants. Figure S5: Breakdown of PathOS only variants not matching public cancer variant annotation resources by analysis type (somatic, haematological and [file 12920_2022_1214_MOESM2_ESM.docx]

# Supplemental Figures


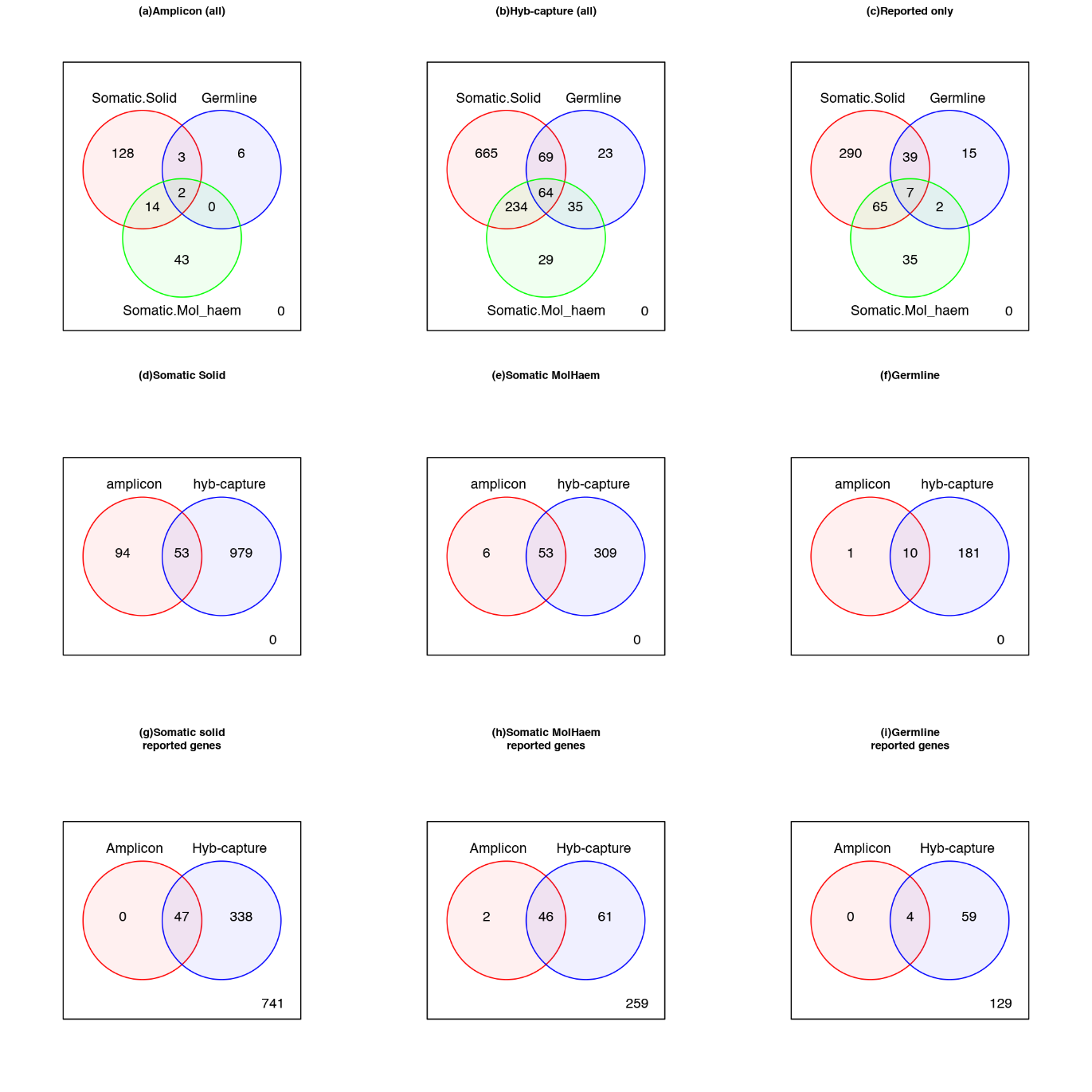
Figure S1: Breakdown of numbers of genes in common across each analysis group and assay. (a) all genes surveyed in amplicon assays, (b) all genes surveyed in hyb-capture
assays, (c) genes containing clinically reported variants only across all assays. (d-f) Displays the genes in common between assays (amplicon and hyb-capture) by analysis type. (g-i) Displays the genes containing reported variants in common between assays by analysis types.

Figure S2: A gene level comparison of consequence between PathOS and VICC. The first column shows the top 20 genes in PathOS. The top row shows the genes coloured by ONC/TSG classification and the black diamond shows the number of distinct variants seen for each gene. The oncogenes have few distinct variants while TSGs and ONC/TSGs have many variants occurring in the gene. This highlights the focal nature of oncogene mutations. The third column shows varinats seen in PathOS after removing corresponding VICC variants showing that common oncogenes appear in VICC but far fewer TSGs and ONC/TSGs.

Figure S3: These graphs show compare the top 20 variant loci between PathOS and VICC. The third graph shows the top 20 variant loci in PathOS after removing matching VICC variants. The predominance of TSG genes becomes apparent.


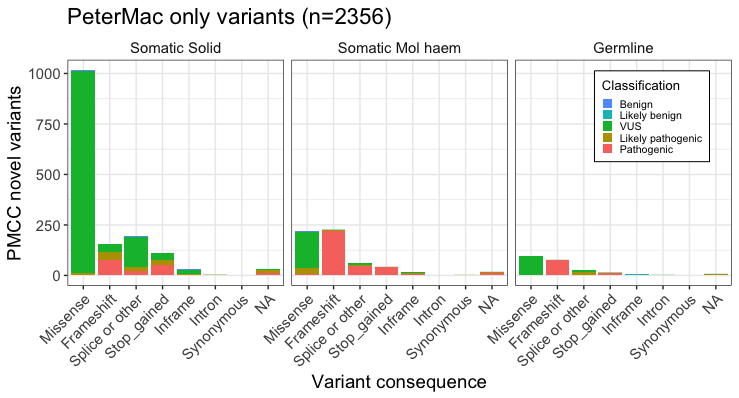


Figure S4: Breakdown of novel variants not matching public cancer variant annotation resources by analysis type (n=2,356). Each variant is classified by functional consequence and coloured by pathogenicity level. Note the high number of somatic, missense, VUS variants.


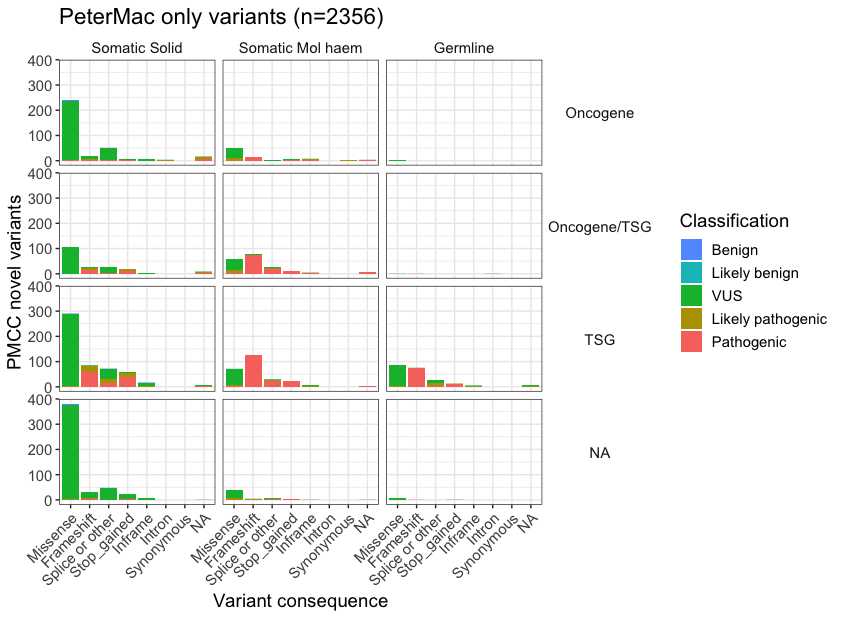


Figure S5: Breakdown of PathOS only variants not matching public cancer variant annotation resources by analysis type (somatic, haematological and germline) (n=2,356). Each variant is classified by functional consequence and coloured by pathogenicity level and separated based on classification of oncogene or TSG.


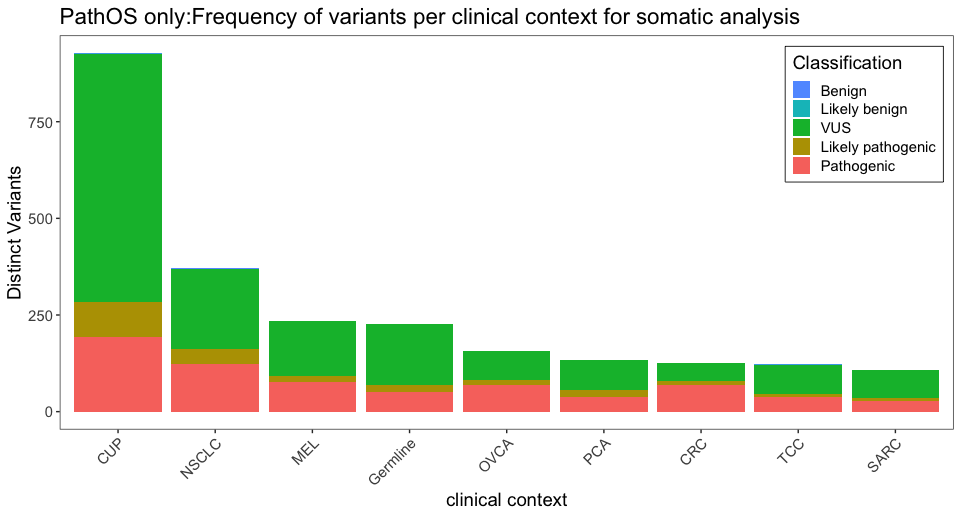


Figure S6: Barplot of somatic solid variants curated by clinical contexts with > 100 variants. CUP=cancer of unknown primary, NSCLC=non-small cell lung cancer, MEL=melanoma, OVCA=ovarian cancer, PCA=prostate cancer, CRC=colorectal cancer, TCC= urothelial carcinoma, SARC=sarcoma.


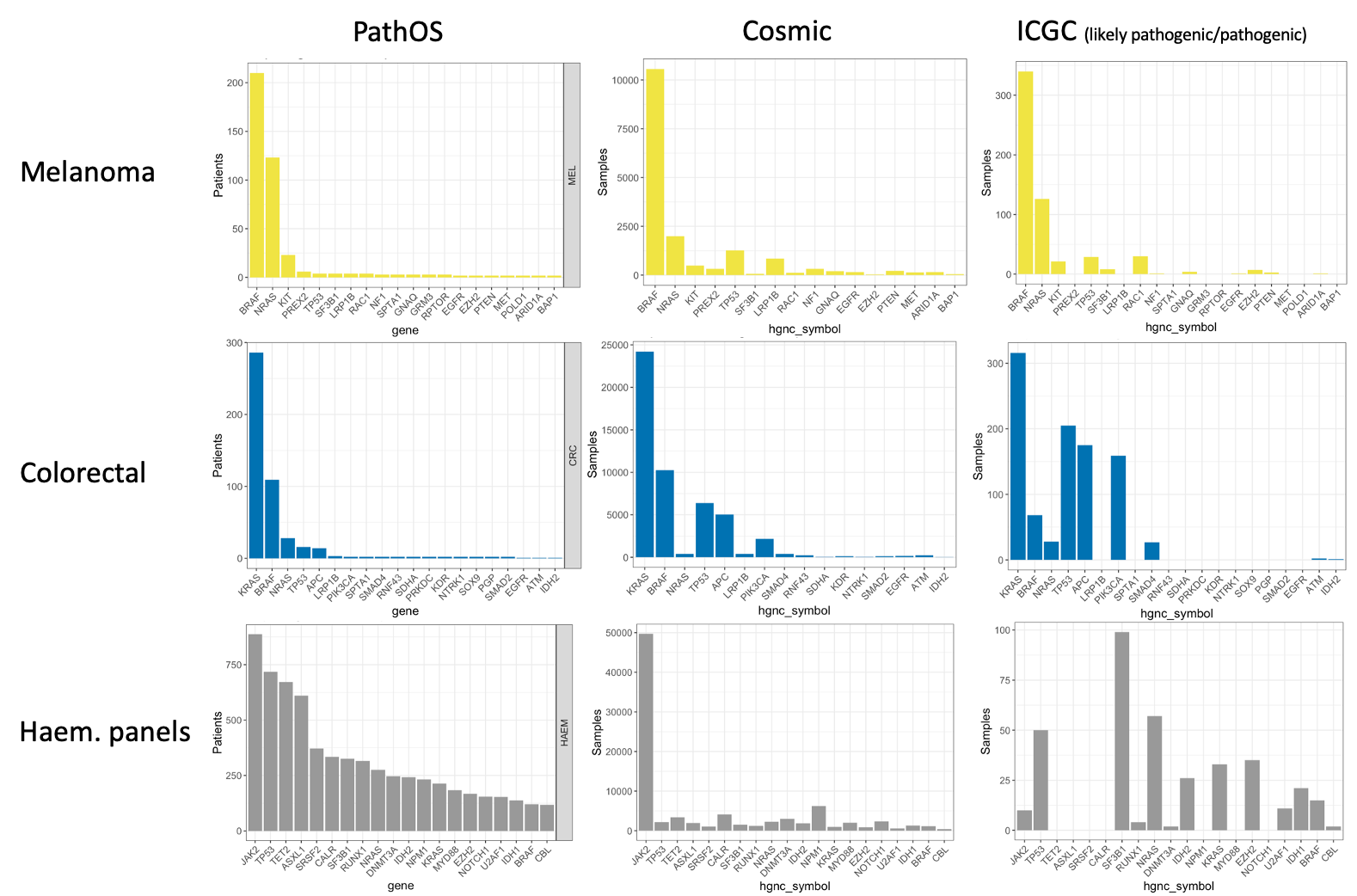


Figure S7 Comparison of patient counts by gene of reported variants between in-house database (PathOS), COSMIC and ICGC for the patient clinical contexts of melanoma, colorectal and haematological malignancies.


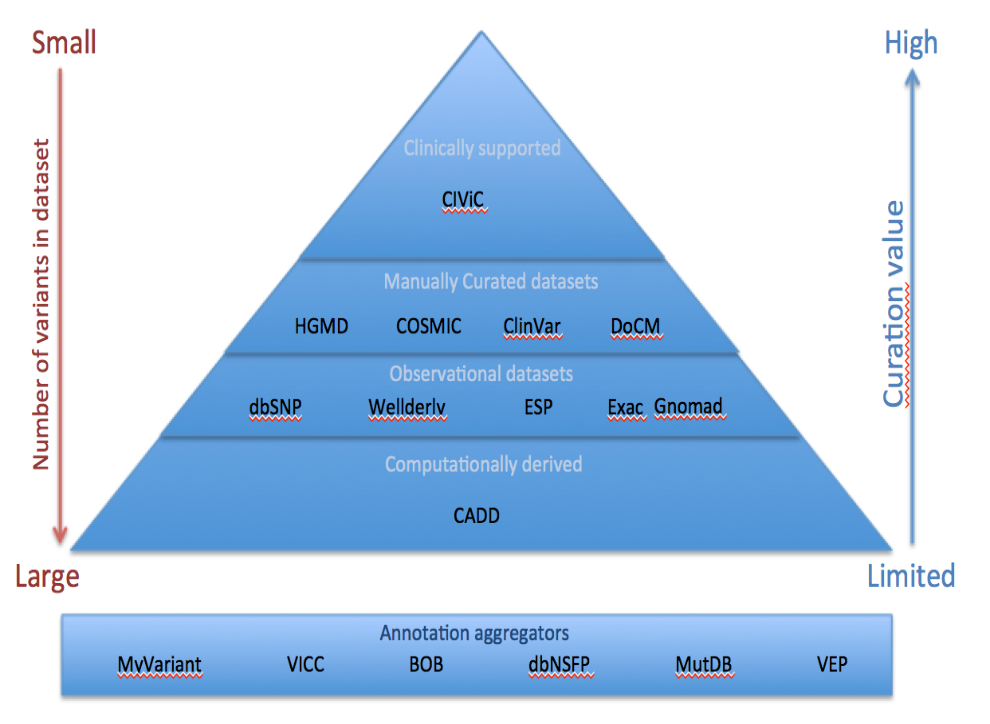


Figure S8: Variant interpretation resources are not all considered equal from a somatic variant curation perspective. Resources with higher curation offer more value than observational or computationally derived resources.


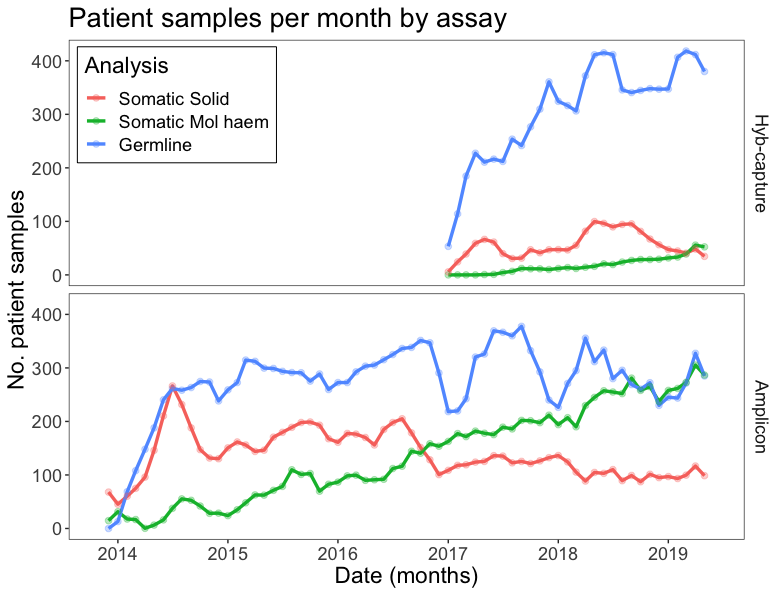


Figure S9: Patient samples analysed per month. A large increase in germline analysis can be observed when hyb-capture assays were implemented in 2017. There is also a steady increase over time in somatic molecular haematology (Mol_haem) samples across both assays. The number of somatic solid samples has remained relatively consistent since 2014 but an increasing number of samples were analysed with hyb-capture assays since 2017. Plotted values are calculated using a three-month rolling average.

# Supplemental Tables

Supplementary file: Panel_summary.xlsx

Table S1: Excel worksheet of panel attributes

Table S2: 24 variants with discordant pathogenicity classifications between CTAP and PathOS

| HGVSg | gene | gene_type | consequence | type | CTAP class | PathOS class | Analysis Group |
| --- | --- | --- | --- | --- | --- | --- | --- |
| chr1:g.45799193dup | MUTYH | TSG | non_synonymous | insertion | VUS | Pathogenic | germline |
| chr11:g.64577385_64577388dup | MEN1 | TSG | non_synonymous | insertion | VUS | Pathogenic | germline |
| chr9:g.139391641G>A | NOTCH1 | oncogene, TSG | non_synonymous_nonsense | substitution | VUS | Pathogenic | haem |
| chr11:g.32413612T>C | WT1 | oncogene, TSG | non_synonymous_splice_site | substitution | VUS | Pathogenic | haem |
| chr11:g.102207666A>T | BIRC3 | oncogene, TSG | non_synonymous_nonsense | substitution | VUS | Pathogenic | haem |
| chr19:g.1612366A>T | TCF3 | oncogene, TSG | non_synonymous | substitution | VUS | Pathogenic | haem |
| chrX:g.133549151G>A | PHF6 | TSG | non_synonymous | substitution | VUS | Pathogenic | haem |
| chr2:g.136872523dup | CXCR4 | oncogene | non_synonymous | insertion | VUS | Pathogenic | haem |
| chr1:g.36932112delinsTC | CSF3R | oncogene | non_synonymous | insertion | VUS | Pathogenic | haem |
| chr4:g.106156113dup | TET2 | TSG | non_synonymous | insertion | VUS | Pathogenic | haem |
| chr4:g.106190901dup | TET2 | TSG | non_synonymous | insertion | VUS | Pathogenic | haem |
| chr11:g.32414215dup | WT1 | oncogene, TSG | non_synonymous | insertion | VUS | Pathogenic | haem |
| chr21:g.36164859dup | RUNX1 | oncogene, TSG | non_synonymous | insertion | VUS | Pathogenic | haem |
| chr21:g.36171755dup | RUNX1 | oncogene, TSG | non_synonymous | insertion | VUS | Pathogenic | haem |
| chr21:g.36259204_36259207dup | RUNX1 | oncogene, TSG | non_synonymous | insertion | VUS | Pathogenic | haem |
| chr1:g.23885707G>A | ID3 | TSG | non_synonymous_nonsense | substitution | VUS | Pathogenic | somatic |
| chr2:g.141250184A>C | LRP1B | TSG | non_synonymous_nonsense | substitution | VUS | Pathogenic | somatic |
| chr3:g.47125785G>A | SETD2 | TSG | non_synonymous_nonsense | substitution | VUS | Pathogenic | somatic |
| chr3:g.47164686G>C | SETD2 | TSG | non_synonymous_nonsense | substitution | VUS | Pathogenic | somatic |
| chr4:g.55144101G>T | PDGFRA | oncogene | non_synonymous_nonsense | substitution | VUS | Pathogenic | somatic |
| chr8:g.90993701A>T | NBN | TSG | non_synonymous | substitution | VUS | Pathogenic | somatic |
| chr9:g.139397732G>T | NOTCH1 | oncogene, TSG | non_synonymous_nonsense | substitution | VUS | Pathogenic | somatic |
| chr12:g.49428235G>A | KMT2D | oncogene, TSG | non_synonymous_nonsense | substitution | VUS | Pathogenic | somatic |
| chr16:g.23641608T>A | PALB2 | TSG | non_synonymous_nonsense | substitution | VUS | Pathogenic | somatic |

Table S3: Correlation analysis of variant recurrence at the gene level between PathOS and publicly available datasets from ICGC and COSMIC, using Pearson’s Correlation Coefficient and log2 scale

| Context | ICGC | | | COSMIC | | |
| --- | --- | --- | --- | --- | --- | --- |
|  | Pearson’s r | P-value |  | Pearson’s r | P-value |  |
| Melanoma | 0.80 | 2.43E-05 | *** | 0.81 | 0.0001 | *** |
| Haem | 0.18 | 0.442 |  | 0.63 | 0.003 | *** |
| Colorectal | 0.74 | 0.0002 | *** | 0.81 | 0.0001 | *** |
